# Scalable salt-templated synthesis of two-dimensional transition metal oxides

**DOI:** 10.1038/ncomms11296

**Published:** 2016-04-22

**Authors:** Xu Xiao, Huaibing Song, Shizhe Lin, Ying Zhou, Xiaojun Zhan, Zhimi Hu, Qi Zhang, Jiyu Sun, Bo Yang, Tianqi Li, Liying Jiao, Jun Zhou, Jiang Tang, Yury Gogotsi

**Affiliations:** 1 Wuhan National Laboratory for Optoelectronics and School of Optical and Electronic Information, Huazhong University of Science and Technology, Wuhan, Hubei 430074, China; 2 Key Lab of Organic Optoelectronics & Molecular Engineering, Department of Chemistry, Tsinghua University, Beijing 100084, China; 3 Department of Materials Science and Engineering and A.J. Drexel Nanomaterials Institute, Drexel University, Philadelphia, Pennsylvania 19104, USA

## Abstract

Two-dimensional atomic crystals, such as two-dimensional oxides, have attracted much attention in energy storage because nearly all of the atoms can be exposed to the electrolyte and involved in redox reactions. However, current strategies are largely limited to intrinsically layered compounds. Here we report a general strategy that uses the surfaces of water-soluble salt crystals as growth templates and is applicable to not only layered compounds but also various transition metal oxides, such as hexagonal-MoO_3_, MoO_2_, MnO and hexagonal-WO_3_. The planar growth is hypothesized to occur *via* a match between the crystal lattices of the salt and the growing oxide. Restacked two-dimensional hexagonal-MoO_3_ exhibits high pseudocapacitive performances (for example, 300 F cm^−3^ in an Al_2_(SO_4_)_3_ electrolyte). The synthesis of various two-dimensional transition metal oxides and the demonstration of high capacitance are expected to enable fundamental studies of dimensionality effects on their properties and facilitate their use in energy storage and other applications.

## Abstract (web summary)

Two-dimensional atomic crystals are known to be effective electrode materials for energy storage applications. Here, the authors report the preparation of various two-dimensional metal oxides, including those which do not have a layered parent structure, via a salt templating strategy.

## Graphical abstract

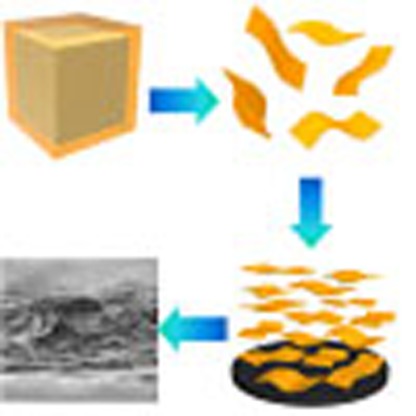

Pseudocapacitors are of great interest for energy storage owing to their fast charging/discharging, high power density and excellent cycling stability, which are beneficial to many potential applications ranging from ubiquitous portable electronics to grid energy storage^1^,^2^,^3^,^4^,^5^,^6^,^7^. Recently, the synthesis of two-dimensional (2D) nanosheets followed by restacking of these nanosheets to form electrodes has attracted much attention^8^,^9^,^10^,^11^. Because of their nanometre/sub-nanometre thickness, nearly all of the metal atoms can be exposed to the electrolyte and potentially involved in redox reactions, resulting in pseudocapacitances that approach the theoretical values^9^. Prominent examples of 2D nanosheets for pseudocapacitor electrodes include MXenes, which have exhibited a volumetric capacitance as high as 900 F cm^−3^ owing to their high conductivity and hydrophilicity, and 1 T phase MoS_2_ nanosheets, which have exhibited a volumetric capacitance of 650 F cm^−1^ (refs 3, 9, 11).

Transition metal oxides, such as MnO_2_ and RuO_2_ are classic pseudocapacitor materials that possess a large theoretical capacity owing to their large number of oxidation states^12^,^13^,^14^. Moreover, oxides have a higher chemical stability than sulfides, carbides and nitrides, such as MoS_2_ (ref. 11) or MXenes^9^,^10^, especially when processed into 2D flakes, which may provide a better durability and enable use in positive electrodes. In addition, many oxides contain tunnels or pores within the crystal structure, which is also beneficial for energy storage applications^2^,^3^. Despite the recent demonstration of promising energy application of nanometre-thick liquid-exfoliated *α*-MoO_3_ (ref. 15) and MnO_2_ (refs 12, 14), the investigation of many other oxides for pseudocapacitor applications is hindered by their lack of a layered structure. Therefore, production of a 2D morphology is challenging.

For pseudocapacitor applications, nanosheets are highly preferable because ion diffusion within the oxide lattice is minimized, enabling the simultaneous achievement of a large volumetric capacitance (high energy density) at a high rate (high power density). This behaviour is typically very challenging to achieve for both bulk and one-dimensional materials. Ideally, the synthesized 2D oxide should be free of contamination from intercalating chemicals, dispersing agents and surfactants that could compromise its electrochemical performance owing to possible blockage of active sites and the added weight/volume of electrochemically inactive contaminants. In addition, the synthesis should be scalable for industrial manufacturing and production of large-size nanosheets with tunable compositions (for example, oxygen vacancies) to compensate for the poor conductivity often encountered in oxides. The current strategies for the production of 2D materials include gas-phase and solution syntheses. Gas-phase synthesis including chemical vapour deposition^16^,^17^,^18^ can produce large-size and high-quality 2D nanosheets on a substrate, but is hindered by a low yield. Solution-based processes, such as liquid exfoliation^12^,^15^,^19^,^20^,^21^ or chemical synthesis^22^,^23^,^24^,^25^,^26^, are simple and scalable. However, these processes lead to small-size particles and have a higher probability of contamination from the synthetic process. One additional limit for exfoliation is its restriction to layered compounds. Only materials bonded by van der Waals or other weak forces in one direction can be easily cleaved by the intercalants. Therefore, the development of a general and scalable strategy for the synthesis of high-quality 2D oxides, including those with non-layered structures and investigation of their potential for pseudocapacitor applications are important.

Herein, we report a general strategy that combines many of the advantages of vacuum-based and solution-based synthetic methods that can be applied to various oxides, including non-layered compounds. Synthesized 2D hexagonal-MoO_3_ (*h*-MoO_3_) is shown to be 300 F cm^−3^ at 5 mV s^−1^ in Al_2_(SO_4_)_3_ electrolyte and approaches the theoretical capacitance of the material at a charge/discharge rate of 2 mV s^−1^ in organic electrolyte. For the 2D oxide synthesis, a molecular precursor solution was first prepared and mixed with a large volume of an inorganic salt (Fig. 1a,b, and Supplementary Fig. 1 shows scanning electron microscopy (SEM) images of precursor@salt templates). Next, the solution was dried and the mixture was annealed at an elevated temperature to produce 2D oxides, through an intermediate hydroxide phase in some cases (Supplementary Fig. 2). The salts were dissolved and the nanosheets were assembled into a restacked film after rinsing and filtration (Fig. 1c,d). In this process, the salt microcrystals served as the substrate/template to guide the oxide growth at elevated temperatures. The as-produced 2D oxides exhibited a large lateral size (up to 100 μm), which is an advantage of a vacuum-based synthesis approach. The process can be scaled up by increasing the amount of inexpensive salt used, as evidenced by the large amount of precursors and final products shown in Fig. 1e and Supplementary Fig. 3. The salt can be dissolved in water, leaving behind clean products that can be further directly filtered into binder- and additive-free films for pseudocapacitor electrodes and other applications (Fig. 1d,f). When integrated into an electrochemical device, ions can easily diffuse from the electrolyte into the restacked film to react with the surface active sites, which are fully exposed in these atomically thin 2D oxides. Under these circumstances, the rate-limiting step of ion intercalation and diffusion in bulk materials is largely circumvented, and all of the active material participates in redox reactions, which contribute to electrochemical energy storage.

## Results

### Synthesis of 2D oxides

Using this strategy, several 2D oxides, including *h*-MoO_3_, *h*-WO_3_, MoO_2_, and MnO were successfully synthesized as shown in Fig. 2. On the basis of the SEM and optical images (Supplementary Fig. 4), all of the samples possess 2D morphology with the size of some flakes exceeding 400 μm^2^, which is much larger than that of liquid-exfoliated or chemically synthesized samples in which sizes are typically limited to a few micrometres^27^. The dimensions of the salt crystals are tens of micrometres and guide the growth of the 2D oxide to a similar size. The transmission electron microscopy analysis of these oxides (Fig. 2e–h; Supplementary Fig. 5) further confirmed the planar 2D morphology, and the high transparency and observed wrinkles suggest that the as-synthesized oxides are nanosheets. All of the oxide crystals had a thickness of <2 nm, which was determined using atomic force microscopy (AFM) (Fig. 2i–l; Supplementary Fig. 6). Selected area electron diffraction, X-ray diffraction (Supplementary Fig. 7) and X-ray photoelectron spectroscopy (XPS; Supplementary Figs 8 and 9) confirmed the formation of *h*-MoO_3_, *h*-WO_3_, monoclinic MoO_2_ and cubic MnO. Moreover, X-ray diffraction characterization also revealed broadened diffraction peaks corresponding to 2D MoO_2_ that were shifted to small angles compared with those of bulk MoO_2_, suggesting lattice relaxation in the 2D MoO_2_ (Supplementary Fig. 10). On the basis of the naturally non-layered crystal structures of these oxides (Supplementary Fig. 11), the suitability of salt-assisted templating as a general method for the synthesis of 2D oxides has been convincingly demonstrated. Despite previous studies using NaCl to synthesize oxide nanocubes^28^,^29^,^30^, to the best of our knowledge, the use of inorganic salts to synthesize 2D oxides has not been previously reported.

### Growth mechanism

Next, we will discuss the synthesis of MnO, which has a non-layered cubic structure (Supplementary Fig. 11d). First, Mn(CH_3_COO)_2_, which was dissolved in ethanol, was used as the molecular precursor to avoid nucleation of oxide nanocrystals in the solution. Second, the thickness of the 2D oxide was controlled by mixing a limited volume of the dilute oxide precursor solution with a large quantity of salt microcrystals. Using this approach, the 2D oxide is believed to be confined to a monolayer thickness, and the formation of structurally stable nanocubes^31^,^32^,^33^,^34^ was avoided because of an insufficient amount of precursor and large-size salt crystals. We measured the size of the salt microcrystals and performed calculations of the precursor-to-salt ratio to meet this requirement (Supplementary Fig. 12; Supplementary Note 1). The AFM analysis indicated that the as-produced 2D MnO crystals were ∼1.3 nm thick (Supplementary Fig. 6d). Third, the crystal geometry of the substrate (salt) should be identical to the target oxide to promote Frank–van der Merwe mode film growth and possible heteroepitaxy^35^,^36^. MnO was cubic with a lattice constant (*a*_1_) of 0.4442, nm. Therefore, KCl was chosen as the template because of its similar cubic structure with a lattice constant (*a*_2_) of 0.3138, nm. Because the crystal symmetry was identical and √2*a*_2_ was 0.4437, nm, overlap of the atomic configuration could be achieved by rotating the crystal plane of MnO by 45°. Figure 3a shows the (001) crystal plane of 2D MnO on the (001) plane of KCl. The mismatch between KCl and MnO is only 0.11% (Supplementary Fig. 13). The X-ray diffraction pattern of the mixture of KCl salt and 2D MnO (after annealing but before rinsing) indicated close pairing of the (200) and (20) diffraction peaks of MnO with the (10) and (200) peaks of KCl, confirming our hypothesis (Fig. 3b). The importance of lattice matching is supported by two facts. First, when Na_2_SO_4_ was chosen as the template salt, no 2D MnO was produced using the same preparation procedure because of the large lattice mismatch between MnO and Na_2_SO_4_ (Supplementary Figs 13e and 14). Second, when we directly dropped the precursor solution onto the ceramic boat (no template) and annealed it under the same conditions, particles were produced rather than 2D structures (Supplementary Fig. 15a,b).

The same approach was used to synthesize 2D *h*-MoO_3_. Mo was dissolved in H_2_O_2_ to produce the molecular peroxomolybdate precursor solution. A precisely calculated amount of precursor was loaded to restrict the growth of thick nanosheets, and NaCl microcrystals were used as lattice-matched templates. Similar close pairing of the diffraction peaks between *h*-MoO_3_ ((10) peak) and the NaCl templates ((20) peak; cubic *a*=5.62 Å) was also observed in the X-ray diffraction patterns (Fig. 3d), and the MoO_3_ crystal growth on NaCl is shown in the inset and in Fig. 3c. As previously suggested in the literature^37^, the formation of *h*-MoO_3_ (*a*=10.584 Å, *c*=3.7278 Å) rather than *α*-MoO_3_ is most likely due to Na^+^ serving as a mineralizer and stabilizer of the hexagonal tunnels in *h*-MoO_3_ in addition to the lattice match-induced *h*-MoO_3_ nucleation. It is important to note that amorphous MoO_3_ particles were obtained when no template was used, further demonstrating the importance of the presence of NaCl (Supplementary Fig. 15c,d). Therefore, we hypothesize that the lateral growth of oxides on the salt surfaces is guided by the salt crystal geometry and promoted by lattice matching, and the growth perpendicular to the salt surface is limited by precursor depletion, which leads to the synthesis of oxide nanosheets with nanometre thickness even for materials with a non-layered crystal structure. In addition, the thickness can be easily controlled by tuning the precursor-to-salt ratio. The thickness increased from 1.6 to ∼12 nm when the precursor-to-salt ratio was increased by a factor of 8 (Supplementary Fig. 16). Nonetheless, more characterizations are required to completely elucidate the mechanism of nanosheet growth on salt templates.

### Electrochemical performance of 2D *h*-MoO_3_

To explore the electrochemical performance of a 2D oxide, 2D *h*-MoO_3_ was selected because MoO_3_ possesses a high theoretical capacity (1.5 Li/Mo)^38^. Notably, this high theoretical capacity is only achievable at a very low current density (0.01 A g^−1^) and a slow charging/discharging rate, which is not suitable for electrochemical capacitors^2^. When processed into 2D nanosheets, we expected that the confinement by slow ion diffusion in bulk MoO_3_ would be relaxed, which would enable fast and reversible surface reactions to achieve high capacitance at high rates to allow for the use of MoO_3_ in pseudocapacitor applications^39^. Using a three-electrode configuration, six different aqueous electrolytes were explored with different cation sizes (Supplementary Fig. 17a,b), and the results are summarized in Fig. 4a. The highest volumetric capacitance of 600 F cm^−3^ was achieved in H_2_SO_4_ at 5 mV s^−1^, which is larger than the value for the Ti_3_C_2_T_*x*_ paper in KOH (∼400 F cm^−3^)^10^ and chemically converted graphene in H_2_SO_4_ (256 F cm^−3^)^8^. All of the results indicated superior rate capability, which is crucial for pseudocapacitors. Surprisingly, the performance in the Al_2_(SO_4_)_3_ electrolyte was 300 F cm^−3^ at 5 mV s^−1^, and to the best of our knowledge, this high capacitance in an Al salt electrolyte has not been previously reported for any material, which opens the door for the use of 2D oxides in Al-ion batteries^40^. Furthermore, the electrochemical impedance spectroscopy results shown in Fig. 4b indicate that the 2D *h*-MoO_3_ electrode exhibited good capacitive behaviour with no apparent charge transfer resistance, which indicates excellent ionic and electronic conductivity. The enhanced electron conductivity arises from the large dimension (fewer and better contact for each flake in the restacked electrode) and abundant oxygen vacancies present in our 2D *h*-MoO_3_ (XPS, Raman spectroscopy, photoluminescence characterizations and conductivity measurements; Supplementary Figs 8a, 18, 19 and 20). These properties are intrinsic advantages of the proposed synthesis strategy where the large size is inherited from the NaCl microcrystal templates and the oxygen vacancies originated from thermal annealing at elevated temperatures in an oxygen-free atmosphere. In addition, a control experiment using ∼12 nm *h*-MoO_3_ nanosheets (Supplementary Fig. 17c,d) resulted in a significantly decreased volumetric capacitance compared with that of the 1.6 nm *h*-MoO_3_ at the same sweep rate, further confirming the effectiveness of nanometre-thin 2D *h*-MoO_3_ to minimize the restriction of ion diffusion^41^. In addition, the electrochemical performance of the MoO_3_ particles that were obtained without templates (Supplementary Fig. 21) was investigated, and a capacitance of <50 F cm^−3^ was achieved, which is significantly lower than that for 2D *h*-MoO_3_ and demonstrates the advantage of the 2D morphology.

Although aqueous electrolytes are widely used, they are inferior to organic electrolytes in terms of the energy density owing to the narrow potential window, which is limited by water splitting. To maximize the energy stored in 2D *h*-MoO_3_, 2D *h*-MoO_3_ was tested in a 1 M LiClO_4_ solution with a 1:1 mixture of ethylene carbonate and dimethyl carbonate. The contact angle measurements indicate that the organic electrolyte provides good wetting of the restacked 2D *h*-MoO_3_ electrode film (Supplementary Fig. 22). According to the cyclic voltammetry (CV) curves at different sweep rates (Fig. 5a), a pair of wide peaks arising from reduction/oxidation reactions contributed to the pseudocapacitance^41^. The charge–discharge curves at different current densities also indicate pseudocapacitive behaviour (Supplementary Fig. 23a). The volumetric and gravimetric capacities are shown in Fig. 5b within a charging/discharging time that ranged from 10 to 1,000 s. Intriguingly, a gravimetric capacity of 996 C g^−1^ (277 mAh g^−1^) at 2 mV s^−1^ was much larger than that previously reported for mesoporous *α*-MoO_3_ in 1 M LiClO_4_ with propylene carbonate (∼580 C g^−1^, 161 mAh g^−1^)^41^. When the charging/discharging time was limited to 10 s, the capacity was still 400 C g^−1^, suggesting that the 2D *h*-MoO_3_ electrode can provide excellent power handling that is suitable for quick charging of portable or wearable electronics.

The redox reaction can be expressed as follows:


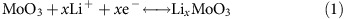


**Figure 1:**
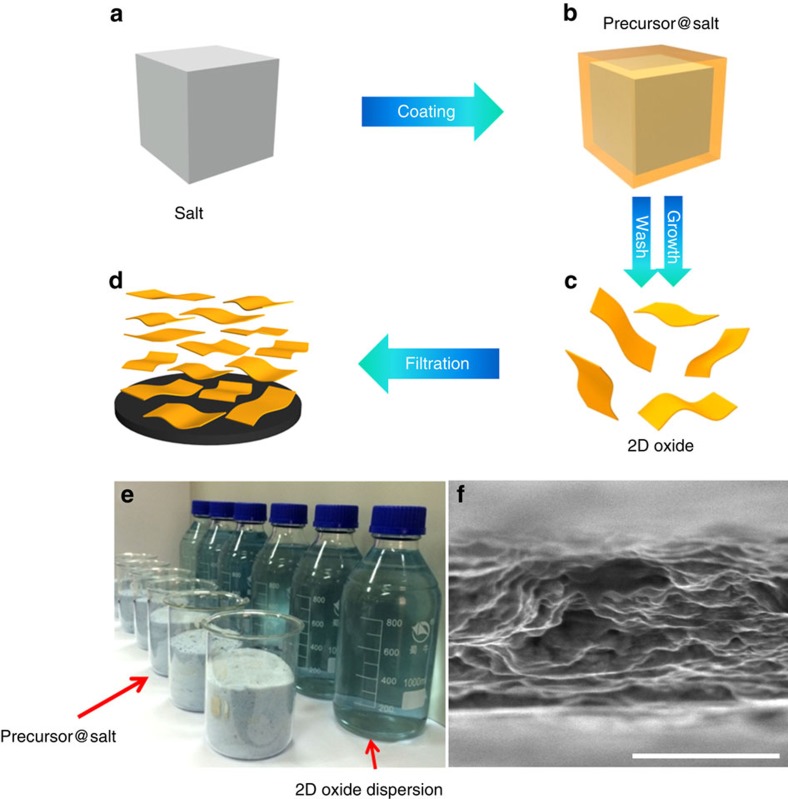
Schematic representation of 2D *h*-MoO_3_ synthesis and electrode fabrication for energy storage. (**a**,**b**) Schematic representation of the fabrication process. The precursor solution was coated on the salt by mixing the solution with a large amount of salt microcrystals. (**c**) After thermal annealing and rinsing in water, 2D oxide flakes were obtained. (**d**) Pseudocapacitor electrodes were fabricated by filtering the 2D oxide/ethanol dispersion using a Celgard separator. (**e**) Optical image of the precursors and 2D *h*-MoO_3_-ethonal dispersions. The front row shows six beakers containing the precursor, and the back row shows six bottles containing the 2D *h*-MoO_3_-ethanol dispersions. (**f**) Multilayered 2D oxide is shown in the cross-sectional SEM image. Scale bar, 1 μm.

**Figure 2:**
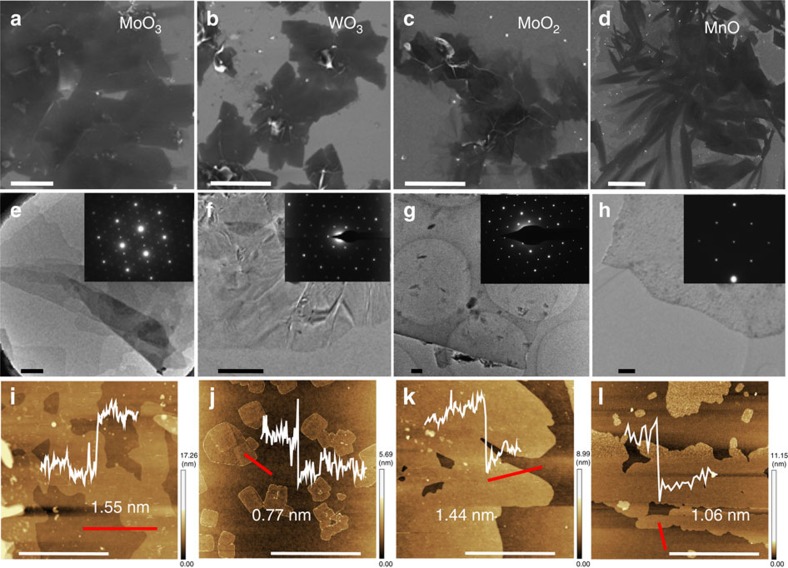
High-quality 2D oxide synthesized using the salt-assisted templating method. SEM images of *h*-MoO3 (**a**) *h*-WO3 (**b**) MoO2 (**c**) and MnO (**d**). Scale bar, 20 μm (**a**,**b**); 100 μm (**c**); 50 μm (**d**). Low-resolution TEM images of *h*-MoO3 (**e**), *h*-WO3 (**f**), MoO2 (**g**) and MnO (**h**). Scale bar, 500 nm (for all of the TEM images). The insets show the corresponding selected area electron diffraction patterns. AFM images of *h*-MoO3 (**i**), *h*-WO3 (**j**), MoO2 (**k**) and MnO (**l**). The insets show the corresponding thickness of the 2D oxide. Scan lines are shown in red. Scale bar, 1 μm (**i**,**k**); 5 μm (**j**,**l**).

**Figure 3:**
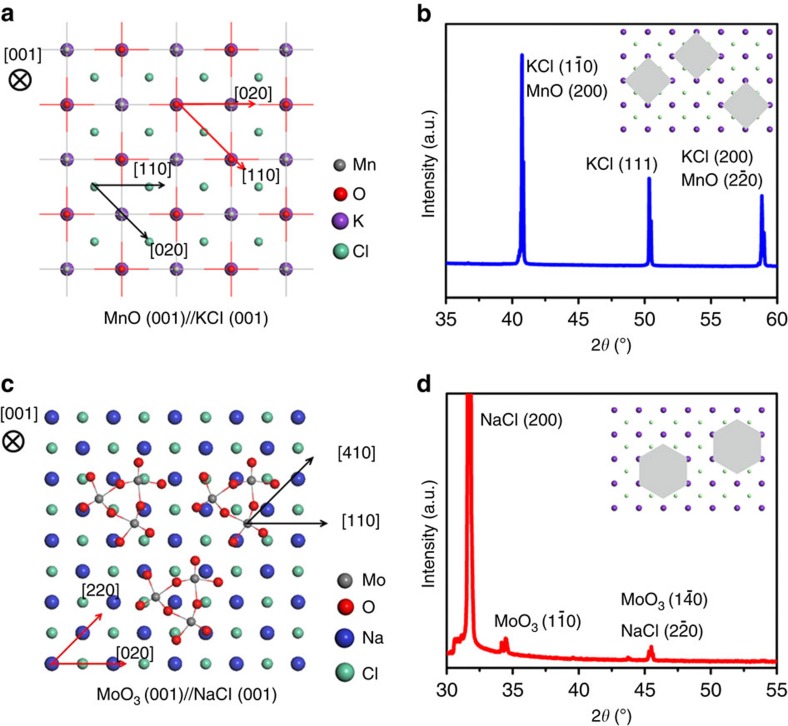
Proposed mechanism of the salt-assisted templated growth of 2D MnO and *h*-MoO_3_. (**a**) Top view of the crystal modes of the MnO (001) plane sitting on the KCl (001) plane. (**b**) X-ray diffraction pattern of 2D MnO on KCl. Please note that the faint diffraction peaks of MnO reside at the left side of KCl diffraction peaks. The inset is a schematic representation of cubic MnO on KCl. (**c**) Top view of the crystal modes of the *h*-MoO_3_ (001) plane sitting on the NaCl (001) plane. (**d**) X-ray diffraction pattern of 2D *h*-MoO_3_ on NaCl. The inset is a schematic representation of *h*-MoO_3_ on NaCl.

**Figure 4:**
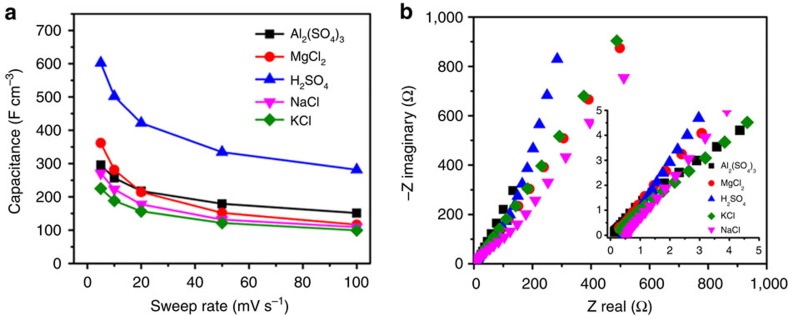
Electrochemical performance of 2D *h*-MoO_3_ in aqueous electrolytes. (**a**) Volumetric capacitance as a function of the sweep rate in different aqueous electrolytes. (**b**) Nyquist plots obtained in H_2_SO_4_, MgCl_2_, NaCl, KCl and Al_2_(SO_4_)_3_ electrolytes.

**Figure 5:**
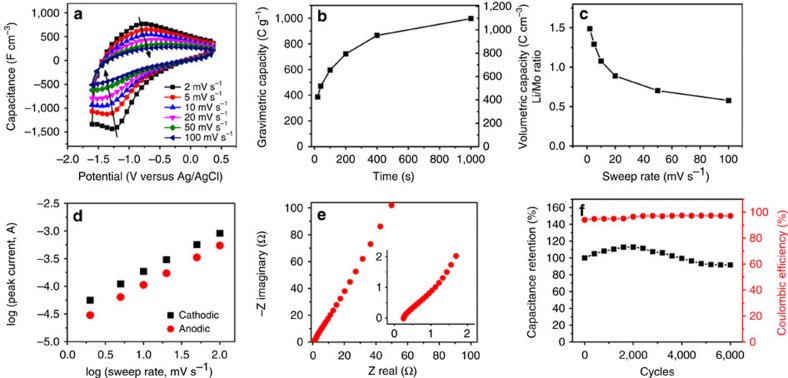
Electrochemical performance of 2D *h*-MoO_3_ in a Li-ion-containing organic electrolyte. (**a**) CV curves at sweep rates ranging from 2 to 100 mV s^−1^ indicating the small peak shifts following the arrows. (**b**) Gravimetric and volumetric capacity as a function of charging/discharging time. (**c**) Li/Mo ratio at different sweep rates using *x*=*QM*/*mF*, where *Q* is the stored charge, *M* is the molecular weight, *m* is the mass and *F* is the Faraday constant. After 1,000 s, 2D *h*-MoO_3_ reaches 1.49 Li/Mo, approaching the theoretical value for bulk *α*-MoO_3_ (1.5). (**d**) Plot for calculating *b* values of the cathodic and anodic peaks (both=0.9). (**e**) Nyquist plots. (**f**) Cycling stability and Coulombic efficiency over 6,000 cycles.

Because the charge of an electron is identical to a lithium ion, the number of Li^+^ involved in the redox reaction can be determined as follows: *x*=*QM*/*mF*, where *Q* is the stored charge, *M* is the molecular weight, *m* is the mass and *F* is the Faraday constant. As shown in Fig. 5c, 1.49 Li^+^ per MoO_3_ were inserted at 2 mV s^−1^ and 0.58 Li/Mo were inserted at 100 mV s^−1^. *α*-MoO_3_ can accommodate up to 1.5 Li/Mo at a very low current density (0.01 A g^−1^), and this amount is largely limited to the surface charge storage at high rates. For bulk and even nanostructured materials with thicknesses greater than the atomic level, slow ion diffusion in solids limits the storage capacity and device power^1^. Restacked 2D *h*-MoO_3_ exhibited a different behaviour because the entire active material was accessible to the electrolyte, allowing for easy Li^+^ transport to the surface that facilitated surface redox reactions with the exposed transition metal atoms and led to a high capacitance even at high sweep rates, as shown in Fig. 1e. Therefore, this high capacitance approaches the theoretical capacitance at 2 mV s^−1^, confirming the advantage of 2D oxides for use in pseudocapacitors.

To gain additional insight into the storage mechanism of the 2D *h*-MoO_3_ electrode, a peak shift of around 0.1 V was studied at sweep rates ranging from 2 to 100 mV s^−1^ (Fig. 5a). This peak shift indicated that the 2D *h*-MoO_3_ was involved in the surface-controlled capacitive behaviour^42^. In addition, the relationship between the sweep rate and peak current was also analysed based on the following equation:


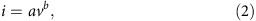


where *i* is the peak current (A), *v* is the sweep rate (mV s^−1^) and *a* and *b* are coefficients^39^. The process is diffusion controlled (battery behaviour) when *b*=0.5 and is capacitive when *b*=1. The *b* values for both the cathodic and anodic peaks were calculated to be 0.9, further supporting the pseudocapacitive storage mechanism (Fig. 5d) that results from the fast and reversible redox reactions of 2D *h*-MoO_3_ with minimal restriction of the ion diffusion. Therefore, a high capacity of 996 C g^−1^ was obtained at 2 mV s^−1^. Electrochemical impedance spectroscopy was also performed, and the results are shown in Fig. 5e. No apparent charge transfer resistance was observed, which indicates a combination of excellent ionic and electronic conductivities in the restacked 2D *h*-MoO_3_ electrodes. The cycling stability of the pseudocapacitor was satisfactory. The initial capacitance increased because of the infiltration of the electrolyte, and 92% of the volumetric capacitance at a sweep rate of 10 mV s^−1^ was maintained after 6,000 cycles (Fig. 5f), which can be further improved by optimizing the electrode architecture. On the basis of the pseudocapacitive mechanism (no phase transformation), these materials are expected to exhibit good cycling stability that exceeds that of Li-ion batteries and simultaneously achieve high power-handling properties and fast charging time.

## Discussion

The most significant result of this work is the demonstrated ability to generate high-quality 2D oxides with various compositions. The successful synthesis of various 2D binary transition metal oxides, combined with their outstanding performance in pseudocapacitor applications, will promote the exploration of 2D oxides for energy storage applications and beyond. In addition, achieving a combination of high gravimetric and volumetric capacitances is important for many applications, such as personal electronics, where the volume is limited. In this study, the volumetric capacity of 2D *h*-MoO_3_ was as high as 1,100 C cm^−3^ at 2 mV s^−1^, which further indicates the advantage of the 2D morphology. Moreover, in order to compare our results with published data for other materials, the volumetric capacitance and energy/power density of the 2D *h*-MoO_3_ in a Li-ion electrolyte are plotted in Supplementary Fig. 23b,c. The highest capacitance value was 550 F cm^−3^, exceeding that for MoS_2_ in 0.5 M 1-ethyl-3-methylimidazolium tetrafluoroborate (EMIM BF_4_)/MeCN (∼250 F cm^−3^)^11^. In addition, the highest volumetric energy density was 270 mWh cm^−3^ with a power density of 1 W cm^−3^.

In conclusion, four transition metal oxides with non-layered structures were successfully synthesized with 2D morphologies using salt-assisted templating. On the basis of our experimental observations, we propose that the lateral growth of the 2D oxides was guided by the salt crystal geometry and promoted by lattice matching, and the thickness was restrained by the raw material supply (controlled amount of the dilute solution). The outstanding pseudocapacitive performance of 2D *h*-MoO_3_ indicates the importance of the 2D morphology in energy storage. This material achieved 300 F cm^−3^ at 5 mV s^−1^ in the Al_2_(SO_4_)_3_ electrolyte, which is favourable for the potential use of 2D oxides in Al-ion batteries. For organic electrolytes, capacities as high as 996 C g^−1^ (1.49 Li/Mo) and 1,100 C cm^−3^ were obtained at a sweep rate of 2 mV s^−1^ owing to fast and reversible redox storage, resulting from fast ion diffusion and electron transport in the restacked 2D *h*-MoO_3_ electrodes. Furthermore, the as-synthesized 2D oxides may find applications beyond energy storage, and this synthetic method could potentially be extended to materials other than oxides.

## Methods

### Synthesis of 2D *h*-MoO_3_ and MoO_2_

In total, 0.1 g of Mo powder was dispersed in 20 ml of ethanol with vigorous stirring for 30 min. Then, 0.35 ml of a H_2_O_2_ (30%) solution was slowly added to the Mo powder suspension, which resulted in a blue solution after stirring for 18 h. Then, the precursor solution was mixed with 320 g of NaCl powder followed by drying at 65 °C with stirring. For *h*-MoO_3_, the as-obtained mixture was further annealed at 280 °C for 1 h at a ramp rate of 2 °C min^−1^ under an Ar atmosphere. After being cooled, the product was washed with deionized water to remove the NaCl templates and dispersed in ethanol for further characterization. For MoO_2_, the same procedure was applied, but the annealing temperature was 550 °C.

### Synthesis of 2D MnO

In total, 0.5 g Mn(CH_3_COO)_2_·4H_2_O was dispersed in 25 ml of ethanol with stirring for 5 min to obtain the MnO precursor solution. Then, the precursor solution was mixed with 400 g of potassium chloride power and dried at 70 °C with stirring. The as-obtained mixture was further annealed at 400 °C for 1 h at a ramp rate of 2 °C min^−1^ under an Ar atmosphere. After being cooled, the product was washed with deionized water to remove the KCl templates and dispersed in ethanol for further characterization.

### Synthesis of 2D *h*-WO_3_

In total, 0.1 g of tungsten powder was dispersed in 20 ml of ethanol with vigorous stirring for 30 min. Then, 1 ml of a H_2_O_2_ (30%) solution was slowly added to the W powder suspension, which produced a white solution after stirring for 36 h. Then, the precursor solution was mixed with 400 g of KCl powder followed by drying at 70 °C with stirring. The as-obtained mixture was further annealed at 500 °C for 1 h using a ramp rate of 2 °C min^−1^ under an Ar atmosphere. After being cooled, the product was washed with deionized water to remove KCl and dispersed in ethanol for further characterization.

### Preparation of electrodes

The vacuum-assisted filtration method was used to fabricate the multilayer electrodes. The as-produced 2D oxide/ethanol dispersion was filtered through a membrane (Celgard, USA). The obtained film was dried at room temperature and then punched into circular electrodes. The thickness of the electrodes was 1 μm. The density of each electrode was ∼1.1 g cm^−3^.

### Characterization

The morphology and structure of the samples were studied using field-emission SEM (FE-SEM, FEI Nova 450 Nano), optical microscopy (Nikon), high-resolution transmission electron microscopy (HRTEM, TECNAI), AFM (Shimadzu), X-ray diffraction (X'Pert Pro, PANanalytical), XPS (ESCALab250), Raman spectroscopy and photoluminescence (LabRAM HR800). For the optical microscopy and AFM measurements, the samples were dropped on SiO_2_/Si and dried at room temperature. The electron conductivity was measured using a Keithley 4,200. All of the electrochemical tests were carried out using ECLab and CHI660E. For the typical three-electrode set-up, Ag/AgCl (CHI, USA) was used as the reference electrode, YP-50 (Kuraray, Japan) was used as the counter electrode and a Celgard film served as the separator (Celgard, USA). All of the tests were conducted in Swagelok cells (Swagelok, USA). The electrochemical impedance was measured from 1 to 1 MHz with a potential amplitude of 10 mV. The organic electrolyte was fabricated by dissolving LiClO_4_ in ethylene carbonate/dimethyl carbonate at a concentration of 1 M.

## Additional information

**How to cite this article:** Xiao, X. *et al.* Scalable salt-templated synthesis of two-dimensional transition metal oxides. *Nat. Commun.* 7:11296 doi: 10.1038/ncomms11296 (2016).

## Supplementary Material

Supplementary Information Supplementary Figures 1-23, Supplementary Note 1 and Supplementary References
