# Supplementary material for: Scalable salt-templated synthesis of two-dimensional transition metal oxides
Source: Nat Commun. 2016 Apr 22;7:11296. doi: 10.1038/ncomms11296 (PMC4844692; doi:10.1038/ncomms11296)
Supplement: Supplementary Information — Supplementary Figures 1-23, Supplementary Note 1 and Supplementary References [file ncomms11296-s1.pdf]

Supplementary Information

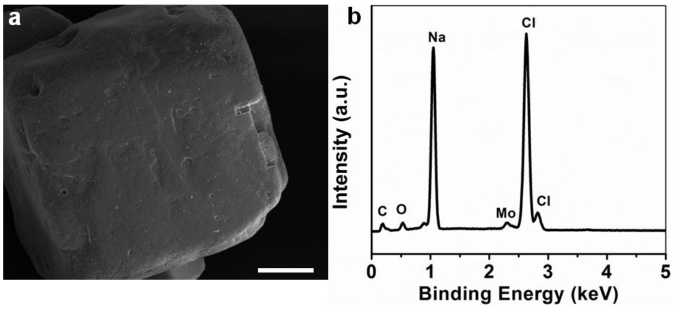

Supplementary Figure 1 | Characterization of precursor coated on salt template. (**a**) SEM image of Mo precursor coated on NaCl. Scale bar, 50 μm. (**b**) EDS of Mo precursor coated on NaCl.

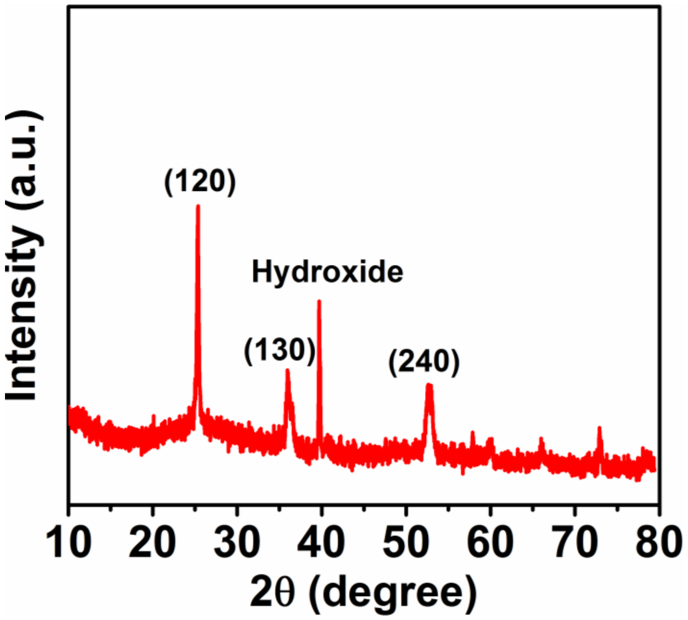

Supplementary Figure 2 | XRD pattern of MoO_3 precursor@salt samples annealed at 280 ℃ for 10 min. The peak at around 40 degree indicates the formation of hydroxide.

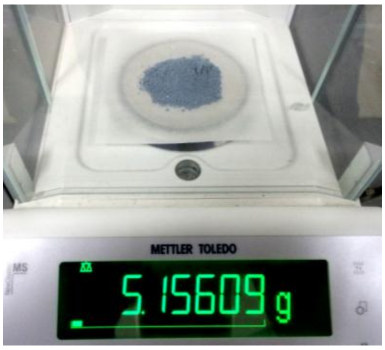

Supplementary Figure 3 | Digital photograph shows the weight of 2D oxide powder.

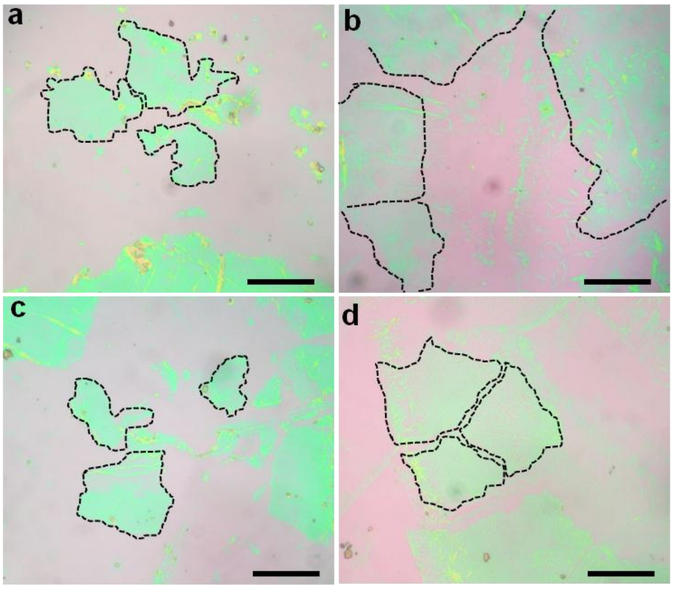

Supplementary Figure 4 | Optical images of transparent 2D oxide flakes. *h*-MoO_3 (**a**), *h*-WO_3 (**b**), MoO_2 (**c**), and MnO (**d**). Scale bar for all of the images, 50 μm.

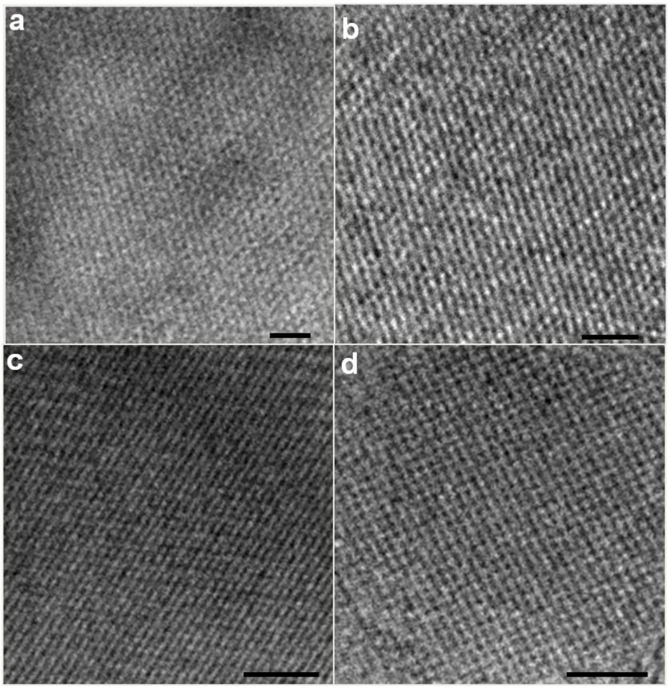

Supplementary Figure 5 | High-resolution TEM images. **a**, *h*-MoO_3, **b**, *h*-WO_3, **c**, MoO_2, and **d**, MnO nanosheets. All of the samples demonstrated a good crystallinity. Scale bar for all of the images, 2 nm.

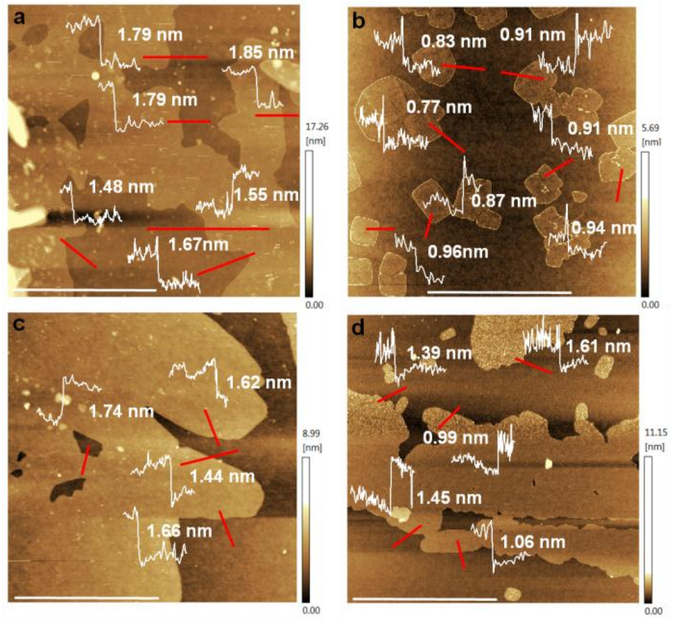

Supplementary Figure 6 | Thickness of 2D oxides measured by AFM. AFM images of *h*-MoO_3 (**a**), *h*-WO_3 (**b**), MoO_2 (**c**), and MnO (**d**). Scan lines are shown in red. Scale bar for **a** and **c**, 1 μm; **b** and **d**, 5 μm.

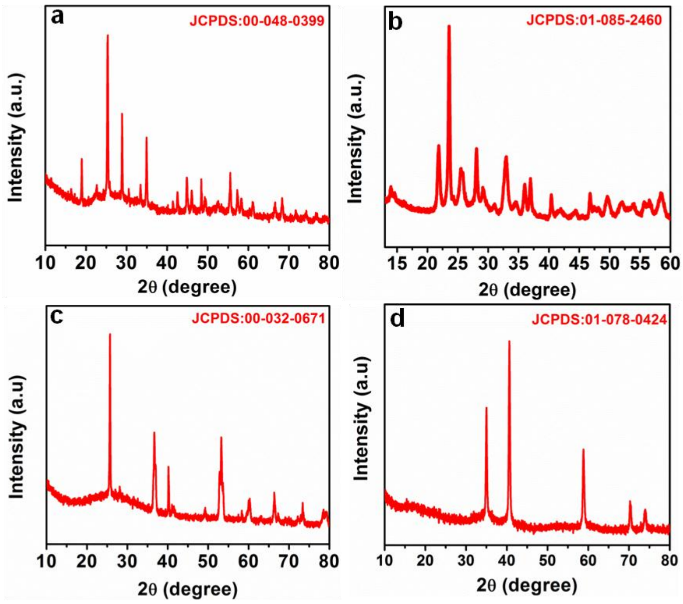

Supplementary Figure 7 | Powder X-ray diffraction patterns of 2D oxides. *h*-MoO_3 (**a**), *h*-WO_3 (**b**), MoO_2 (**c**) and MnO (**d**).

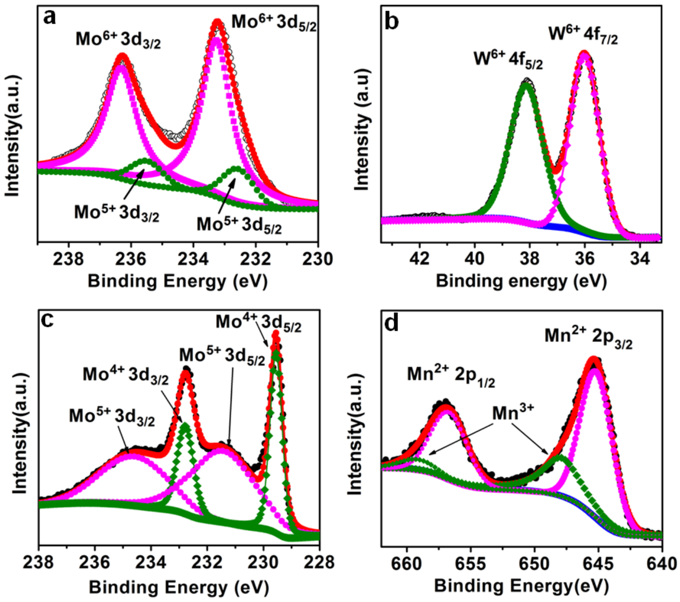

Supplementary Figure 8 | XPS spectra of 2D oxides. *h*-MoO_3 (**a**), *h*-WO_3 (**c**), MoO_2 (**d**), and MnO (**b**).

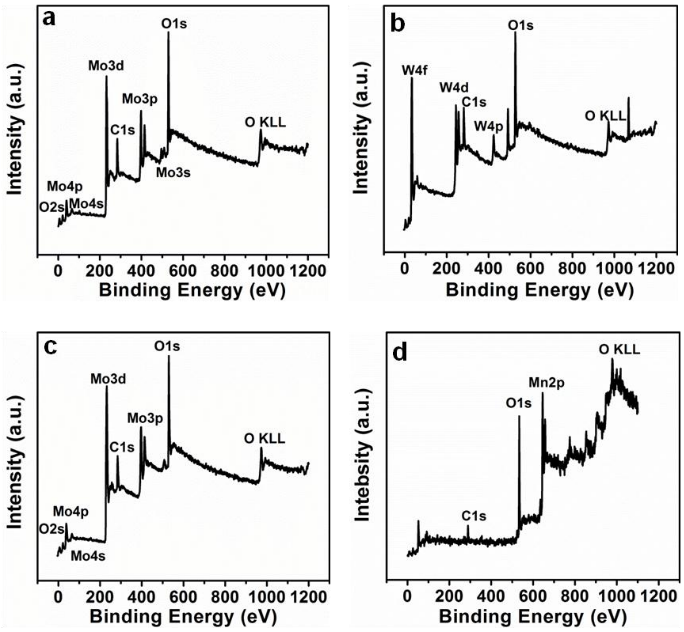

Supplementary Figure 9 | XPS survey spectra. (**a**) *h*-MoO_3, (**b**) *h*-WO_3, (**c**) MoO_2, and (**d**) MnO samples.

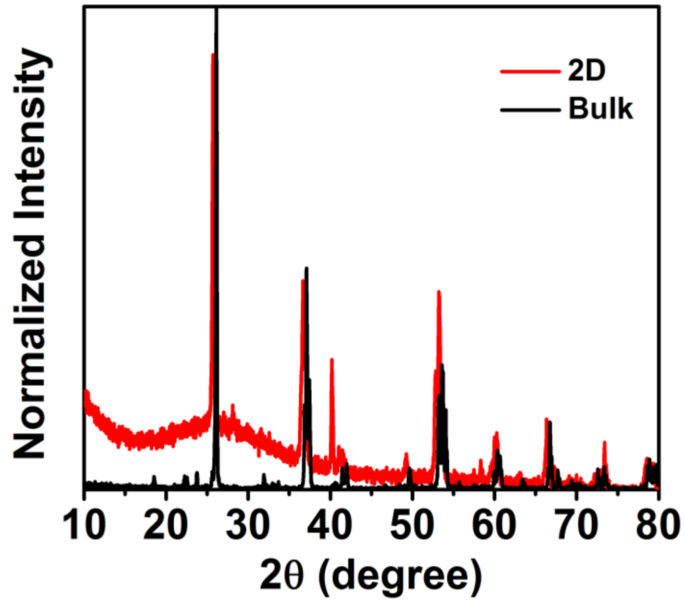

Supplementary Figure 10 | XRD patterns of 2D MoO_2 and bulk MoO_2.

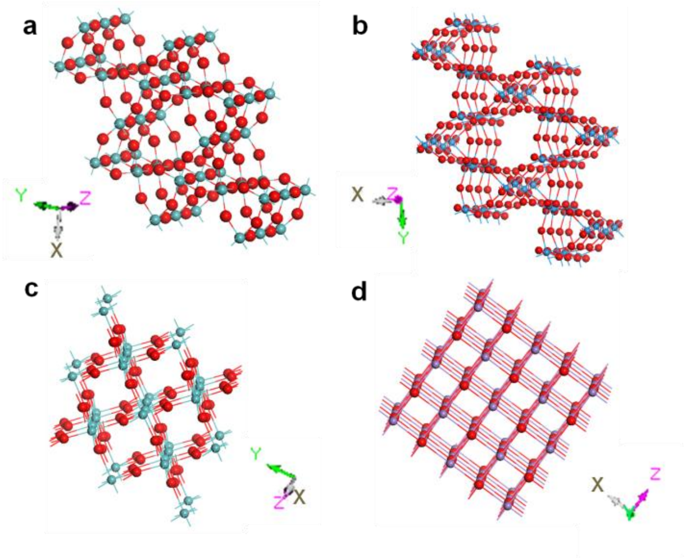

Supplementary Figure 11 | Crystal structure of 2D oxides. *h*-MoO_3 (**a**), *h*-WO_3 (**b**), MoO_2 (**c**), and MnO (**d**).

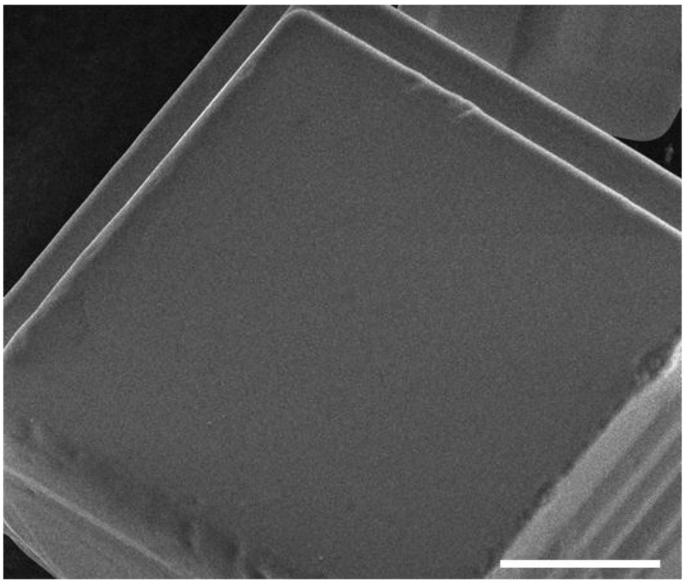

Supplementary Figure 12 | SEM image of a KCl microcrystal. Scale bar, 20 μm

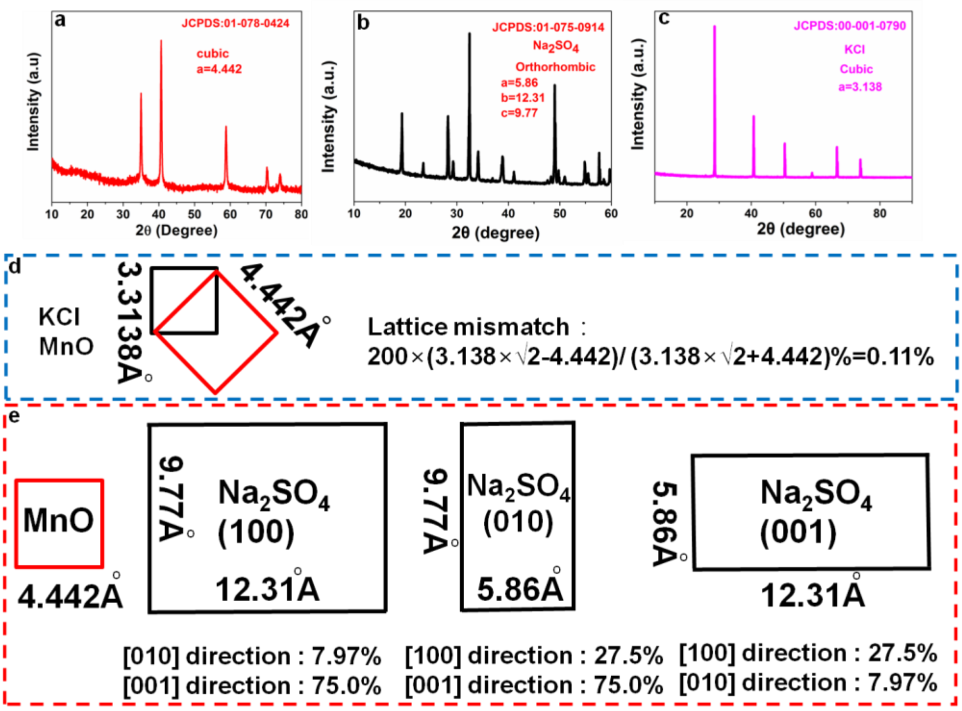

Supplementary Figure 13 | Growth mechanism. a~c, XRD patterns of MnO, Na_2SO_4 and KCl. **d**, Lattice mismatch of MnO and KCl, differing by only 0.11%. **e**, Large lattice mismatch of MnO and Na_2SO_4, no matter the direction of measurement. This is the reason why Na_2SO_4 cannot be used as the template to synthesize 2D MnO.

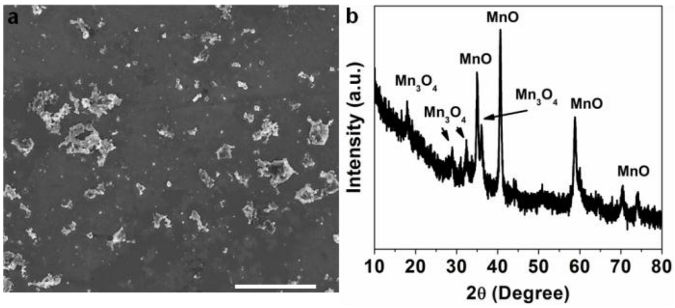

Supplementary Figure 14 | SEM image and XRD pattern of MnO and Mn_3O_4 particles which were synthesized by using Na_2SO_4 as templates. When using Na_2SO_4, no 2D MnO nanosheets were found. Scale bar, 30 μm.

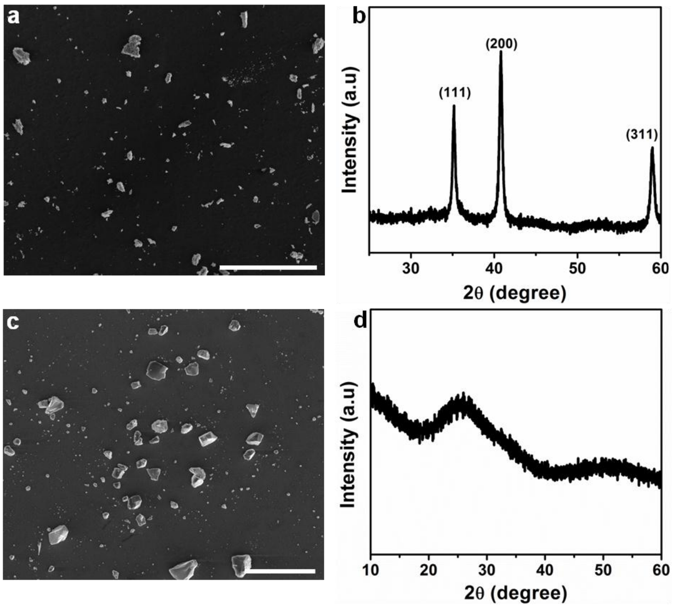

Supplementary Figure 15 | SEM image and XRD patterns of MnO and MoO_3 grown without templates. a~b, SEM image and XRD of MnO without templates. Scale bar, 300 μm. **c~d**, SEM image and XRD of MoO_3 without templates. We dropped the precursor solution on the ceramic boat and annealed them with the same condition of 2D oxide. Scale bar, 50 μm.

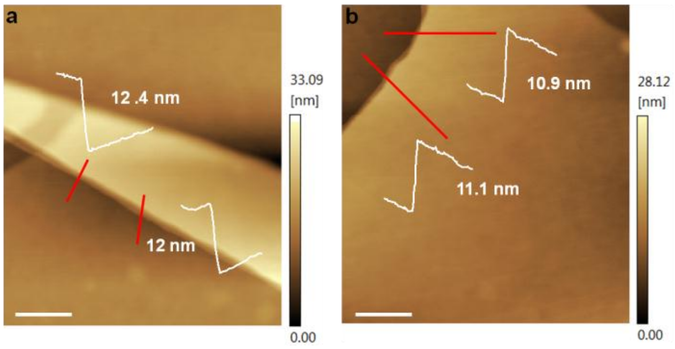

Supplementary Figure 16 | AFM images of thick 2D *h*-MoO_3 flakes. The thickness could be easily tuned from ~1 to ~12 nm. Scale bar, 200 nm.

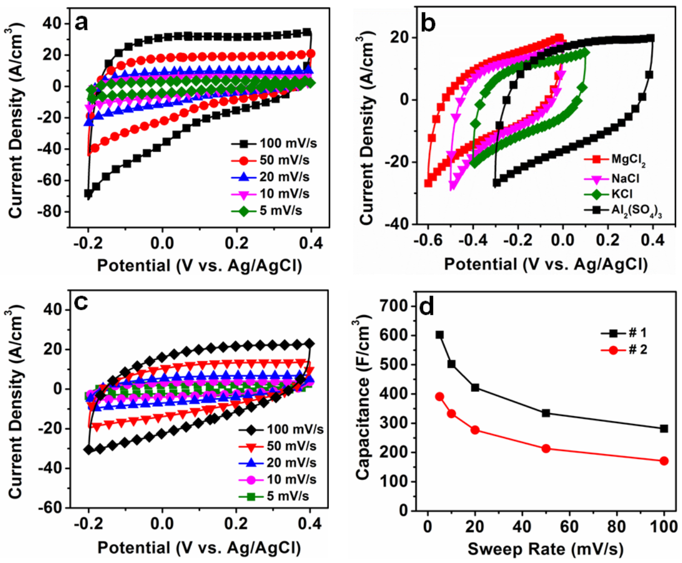

Supplementary Figure 17 | Electrochemical performance of 2D *h*-MoO_3 in aqueous electrolytes. a, CV curves from 5 to 100 mV/s in 1 M H_2SO_4. **b**, CV curves in different electrolytes ($MgCl_2$, KCl, NaCl and $Al_2(SO_4)_3$) at 100 mV/s. **c**, CV curves of thick *h*-MoO_3 from 5 to 100 mV/s in 1 M H_2SO_4. **d**, Volumetric capacitance versus sweep rate for 1 nm and 12 nm 2D *h*-MoO_3. #1 is 1 nm 2D *h*-MoO_3 and #2 is 12 nm 2D *h*-MoO_3, respectively.

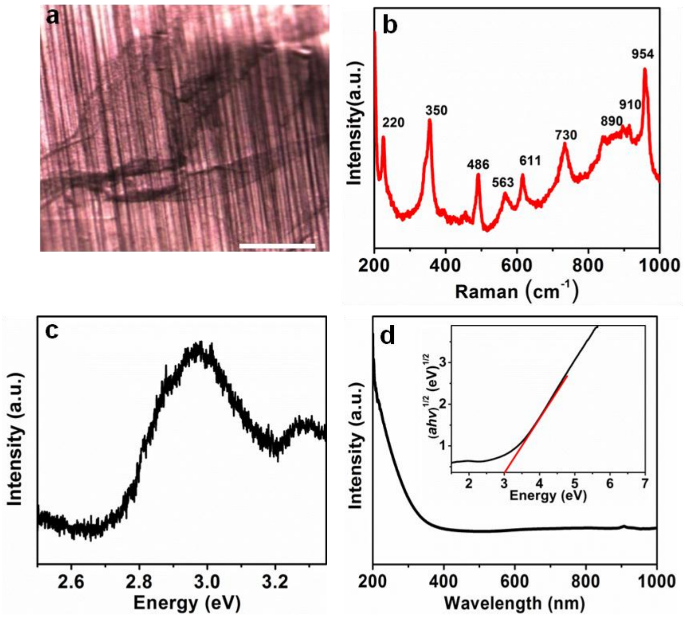

Supplementary Figure 18 | Characterization of 2D *h*-MoO_3. **a**, The optical microscope image of a *h*-MoO_3 nanosheet used for PL and Raman testing. Scale bar, 30 μm. **b**, Raman spectra for as-prepared *h*-MoO_3 nanosheet acquired with using the 532 nm excitation wavelength. The presence of 730, 890, and 954 cm^{-1} bands is pointing toward a change in coordination spheres around Mo centers, which also suggests that octahedra (954 cm^{-1}) in our samples are less distorted than in pure MoO_3. In another words, there are oxygen vacancies in our sample.[1] **c**, The room temperature photoluminescence spectrum of as-prepared *h*-MoO_3 nanosheet samples exited with wavenumber 325 nm. A broad peak at about 2.96 eV is observed in the range of 2.8-3.2 eV, and another shoulder peak is centered at 3.28 eV in the range of 3.2-3.4 eV, respectively. The band at 2.96 eV is attributed to the intrinsic nature of the MoO_3 single crystal, whereas the 3.28 eV band might be due to imperfections of the lattice; namely, the oxygen vacancies caused by the deficiency of oxygen. **d**, UV-Vis absorption spectrum of as-synthesized *h*-MoO_3 nanosheets. The inset is the plot of $(ahv)^{1/2}$ vs photo energy (h*v*) for the *h*-MoO_3 nanosheets.

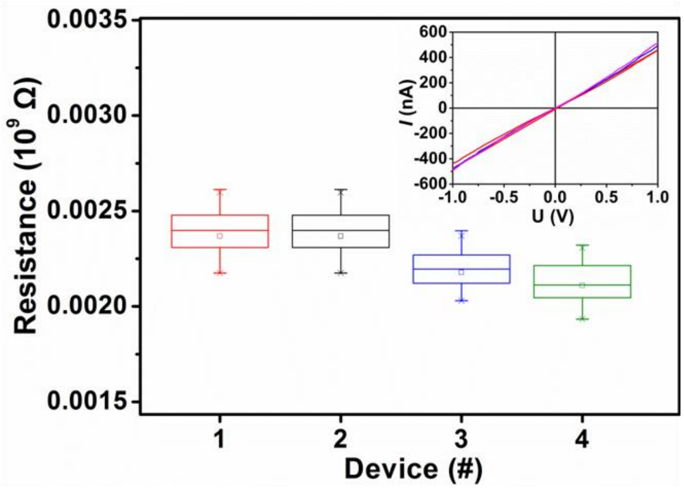

Supplementary Figure 19 | Resistivity of the 2D *h*-MoO_3 electrode. The inset is the I-V curve. We used masks (10 μm tungsten wire as grids) to fabricate electrode, after that, Au electrodes with 80 nm thick were patterned on samples by thermal evaporation. The conductivity is calculated to be 0.02 *S*/m.

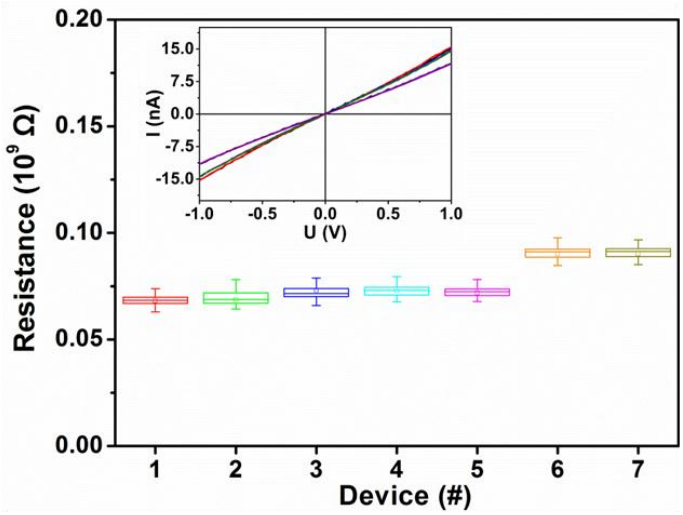

Supplementary Figure 20 | Resistivity of a single 2D *h*-MoO_3 nanosheet. The inset is the I-V curve. 2D *h*-MoO_3 were dropped on SiO_2 (300 nm)/Si substrate and drying at room temperature. The masks used here were fabricated by using 10 μm tungsten wire as grids, after that, Au electrodes with 80 nm thick were patterned on samples by thermal evaporation. The conductivity is calculated to be 1.19 *S*/m.

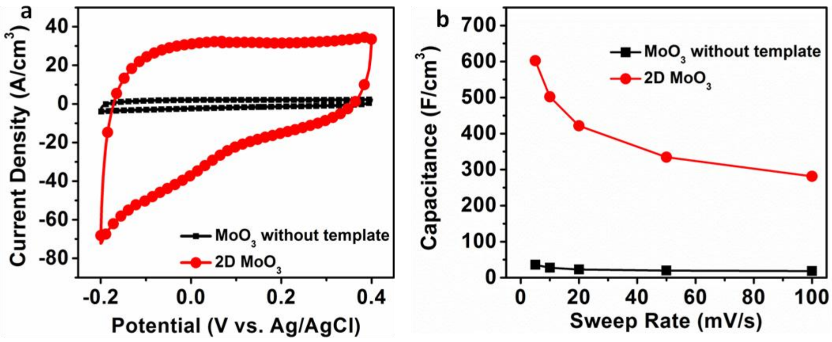

Supplementary Figure 21 | Electrochemical performance comparison of 2D *h*-MoO_3 with MoO_3 grown without templates. a, CV curves of 2D *h*-MoO_3 and MoO_3 without templates. **b**, Capacitance vs. sweep rate for different samples.

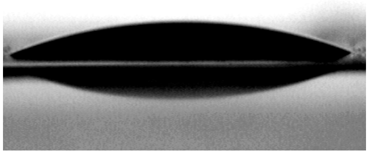

Supplementary Figure 22 | Wetting of 2D *h*-MoO_3 paper with organic electrolyte. 25 ° contact angle of organic electrolyte (1M $LiClO_4$ in EC/DMC) on 2D *h*-MoO_3 paper.

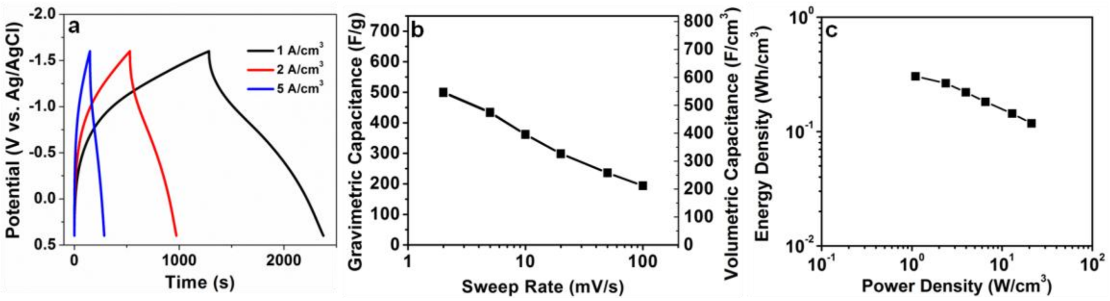

Supplementary Figure 23 | Electrochemical performance of 2D *h*-MoO_3 paper in a Li-ion electrolyte. **a**, Charge-discharge curves. **b**, Gravimetric and volumetric capacitance versus sweep rate. **c**, Ragone plot.

Supplementary Note 1. **Calculation of the thickness of 2D MnO nanosheet**

Assuming: i) 100% coverage of MnO precursor on KCl microcrystal surface; and ii) no evaporation of MnO during the annealing process. Terms (i) and (ii) are ideal conditions because 100% coverage is unlikely and significant evaporation would occur due to the large vapor pressure of MnO. Therefore, this calculation is used as an estimate to guide experiments.

For the calculation, the average size of the KCl microcrystals was measured by SEM to be 100 μm. For the synthesis, 400 g KCl microcrystals were mixed with 20 ml $Mn(CH_3COO)_2$ precursor solution containing 0.145g MnO, proceeded by the following calculation:

$\rho_{KCl} = 1.984\ g/cm^3$ (1)

$\rho_{MnO} = 5.40\ g/cm^3$ (2)

Mass of a single KCl grain = $\rho \times V = 1.984\ g.cm^{-3} \times (1\times10^{-2} cm)^3 = 1.984\times10^{-6}\ g$ (3)

The number of KCl grain = $400\ g/(1.984\times10^{-6}\ g) = 2.016\times10^8$ (4)

A(surface area) = $2.016\times10^8 \times 6 \times (1\times10^{-2}\ cm)^2 = 120960\ cm^2$ (5)

Assuming MnO covered on all the KCl microcrystals with identical thicknesses: A*h (thickness) = $V_{MnO} = m_{MnO}/\ \rho_{MnO}$ (6)

$m_{MnO} = 0.145\ g$ (7)

$V_{MnO} = 0.145\ g/(5.40\ g.cm^{-3}) = 2.69\times10^{-2}\ cm^3$ (8)

$h = V/A = 2.69\times10^{-2}\ cm^3/120960\ cm^2 = 2.424\times10^{-7} cm = 2.224\ nm$ (9)

***Supplementary References*:**

1. Miyoshi, A., Tsuchiya, K., Yamauchi, N. & Matsui, H. Reactions of Atomic Oxygen (3P) with Selected Alkanes. *J. Phys. Chem.* **98**, 11452-11458 (1994).
